# Remarks on Iodine, Read before the Medical and Philosophical Society of Cincinnati

**Published:** 1830

**Authors:** Landon C. Rives


Art.—III. *Remarks on Iodine, read before the first Medical District Society of Ohio.* By Landon C. Rives, M. D.

The energetic action of iodine upon the human system, the salutary influence which it has manifested in many cases of the most obstinate morbid affections, and the prospect that the sphere of its utility may yet be enlarged, by bringing under its dominion other diseases which now very generally resist the established modes of treatment, constitute for it a title to professional interest and enquiry, which, few articles of the materia medica, possess at this time in an equal degree.—Numerous and exceedingly interesting experiments have been made with this active agent by physicians both in Europe, and in this country—the results, however, so far as we know, have by no person been collected or embodied. The attempt will constitute the scope and design of the present paper, and if in the progress of our research, we should be enabled to make a fair estimate of its value, or to deduce any practical precept which should guide us in its administration, or direct us in our future experiments with it, we shall be compensated for the labour incident to the undertaking.

The credit of introducing iodine into the practice of medicine is due to Mons. Coindet of Geneva. In the year 1820 he announced it as a valuable remedy in Goitre. What were the considerations which influenced him to experiment with the article, we have no means of determining ; but his success was such as to draw to it, immediately, the notice of the profession. In his first trials, the tincture of iodine only was used, but having observed occasionally certain unpleasant symptoms to arise which he attributed (erroneously, we think) to its internal administration, frictions with the ointment of hydriodate of potash were substituted by him in his subsequent practice, and as he avers with the same decided influence upon the disease, and without any of those untoward symptoms which accompanied its internal use. Of twenty three cases which he reports to have managed by inunctions alone, twelve were entirely cured and the residue were all benefitted. The favourable report of Coindet is fully sustained by the testimony of Coster, Breschet, Gunther, De Carro and a host of other experimentalists on the continent of Europe. By the first named physician, we are told that of "nearly 100 persons whose cases he had collected, more than two thirds were completely cured by it," and we do not believe that the average proportion of cures would be essentially varied, (we are sure, it would not be diminished) if all the cases which had ever been treated by it, could be collected. The experience of the physicians of Great Britain and America, which has accumulated rapidly in a few years past, tends to corroborate the statements from the continent. We are therefore, after a careful comparison of the reports of the profession generally—prepared to accord to iodine the merit of being the most efficient remedy in Goitre, with which we are acquainted. We would be far however, from encouraging an expectation of uniform success. Our experience reprobates the idea of a specific, and the very name is abhorrent, in our view, to all the principles of a correct medical philosophy. In many instances we are satisfied that iodine has failed to produce any diminution in the volume of goitrous tumours, though persevered in, and given in as large doses as the stomach and strength of the patient would allow. These instances however, are comparatively few, and they would probably be still farther reduced in number, if the medicine were judiciously aided by other means. If the tumour be inflamed, emollient fomentations and the local detraction of blood by leeches, are valuable subsidiary agents, and if it be hard and indolent, electricity should be super-added. M. Coster's method of using this latter remedy, being peculiar, deserves to be stated. It is known that the positive pole of a galvanic pile exercises an attractive action on iodine. With this fact before him, M. Coster conceived the idea that "by making use of frictions with pure iodine on one side of the tumour," and alternating it at each application and "applying the pole on the opposite side, the absorption would be more speedy, and the effects of the iodine on the tumour more sensible." We are not prepared, from the single case which he presents in illustration of his suggestion, to attach to it any great importance. It nevertheless deserves to be remembered. Instances of diseases yielding to the conjoint action of remedies, which, when separately employed had been unsuccessful, are by no means unusual in the practice of medicine ; and this case, in our opinion, only presents another example of the fact. The iodine and electricity awakened the activity of the absorbents which, either unaided by the other, had been too feeble to accomplish.

In pursuing the history of our medicine, we find that the success which attended its employment in Goitre, very soon suggested experiments with it in scrophula, and although from a comparison of all the medical testimony we have been enabled to collect, it does not appear to exert so decided an influence in this as in the former disease, there is abundant and undoubted evidence to satisfy us that it deserves to rank among the most important of our anti-strumatic remedies. To this point we have the testimony of Kolley, Garidner, Gocden, Bardsley and others. But let us not imagine that this or any other medicine is endowed with properties to meet all the various indications which are presented to us in the progress of this complex and pervading disorder. From a disposition to hunt after specifics we are prone to overlook the conditions of the system which controul or modify the action of medicinal agents, and to expect from one, what can only be achieved by the skilful management of many medicines. Much difficulty has attended every attempt to illustrate the origin, nature, and treatment of scrophula, and the whole subject is even now involved in obscurity and uncertainty. It is doubtless, an hereditary disease, though it is also often acquired. It is associated with a prominent developement and irritability of the lymphatic system, and by most pathologists, is supposed to have its primary seat in this system. We doubt however very much the correctness of this view of the disease. Accustomed in all of our pathological speculations to give to the digestive organs, an important agency in the production and propagation of diseased action, we have found strong reason in our limited experience with this, to assign to it the same location. We have been led to adopt this view of its pathology, from a consideration of its symptoms, its causes and its treatment.

Among the earliest phenomena of the discase, if indeed they be not the very first, are variable or deficient appetite, nausea, irregular bowels, often constipated, flatulence and precordial oppression. So prominent are these symptoms, that they are universally present in every case of scrophula, and are recognized long anterior to the *developement* of the disease, as intimately associated with the strumous diathesis. If we pass from the symptoms to the causes of scrophula, we shall find that the latter equally declare its origin to be in a disordered state of the chylopoietic viscera. Had we time to engage in a separate analysis of each, we are persuaded, that it would be an easy task to shew that they all might be resolved into such, as impair, directly or indirectly the powers of the stomach. We have only to designate the most operative of them, to make it manifest that such is their mode of action. Unwholesome aliment, a damp and close atmosphere, intestinal worms, the abuse of opium, of heat and of cold, and the want of cleanliness and of exercise, are the most productive causes of scrophula. Are they not also, those which are best calculated to weaken the powers of digestion ?

Our position is moreover sustained by a view of the prophylactic, and curative treatment of scrophula. The latest and most authoritative writer, on the disease declares that "*dietetics* is omnipotent in its prevention," and again that "*dietetics* and physical education are the only means capable of eradicating the scrophulous taint." For these reasons we cannot assent to that theory of this disease, which radicates it in the lymphatic system. We have already admitted, that there is connected with the strumous diathesis, a peculiar irritability of the lymphatic system, which renders it more susceptible to the morbific influence of irritants. But this condition being congenital and frequently compatible with the most perfect health, is, we conceive distinct from disease itself. We take leave of this digression, by repeating our conviction that the primordial derangement in scrophula, exists in the organs subservient to the processes of digestion, assimilation and nutrition; that commencing in the stomach, it is extended to the bowels, to the lacteals, to the mesenteric glands, and finally to the lymphatics which is the seat of its most obvious and prominent symptoms.

Scrophula is marked by two very distinct stages which exact a modification of remedies. In the first or occult, the treatment resolves itself into one of a tonic and corroborant character. Occasional purging, tonics, exercise, regimen and bathing, constitute the sum of what should here be attempted—but when the disorder is fully developed, it calls for a much more diversified treatment. Purging is then required, not only to remove alimentary accumulations and vicious secretions, but to overcome the inflammatory diathesis which frequently accompanies this stage of the disorder. With the same view of reducing the action of the blood vessels, it is often necessary to resort to the lancet, and the anti-phlogistic treatment in all its details. These means judiciously combined with tonics, narcotics and local remedies will frequently accomplish cures, even in this stage ; but in protracted cases, and more especially in such as are accompanied by an affection of the skin and eyes, it has been usual to resort to mercury. Exactly under such circumstances, I presume, iodine is most useful and should be preferred to mercury. It is generally conceded that a decided mercurial impression is mischievous in this disorder, by exciting irritation and debility, and it is also well ascertained that mercury does not act with energy upon the absorbents until this point is attained. We appeal for the truth of this remark, to its operation in ascites. Repeatedly have we seen the ordinary combination of squills and calomel prove inert, until its effects were displayed on the salivary glands, when immediately absorption would commence and go on rapidly. Now in iodine, we have an agent which will act with as much energy upon the absorbent system, whilst upon the stomach it displays a tonic action , thus fulfiling two most important indications in scrophula. Iodine is perhaps less irritating also, and hence may be used in forms of the disorder, in which mercury is absolutely mischievous. In tuberculous lungs, the best authorities concur in reprobating the use of mercury, the effect of every preparation of this mineral, being invariably, to excite the tubercles into action, and to hasten their suppuration. Such is not, however, the action of iodine. Dr. Berton's experiments with the vapour of iodine, in incipient consumption, would lead us, on the contrary, to expect benefit from it. In two, of the three cases he reports, the cough and expectoration were greatly relieved, and the appetite improved. These results with the later testimony of Dr. Baron, who is sanguine that he has succeeded in dispersing tubercles of the lungs by this remedy" and the negative testimony of Mons. Lugol, who declares that he has never known it to prove injurious to pulmonary tubercles or hæmoptysis, should incite us to further trials with it. But in this deplorable malady, I confess, that I indulge very little expectation of benefit from iodine or any other medicinal agent. Its unhappy subject will find his best hope of a prolonged existence in a temperate and abstemious regimen, in a mild and uniform climate, in moderate exercise and in an avoidance of all those causes which are calculated to produce vascular excitement. When once disorganization has commenced, all the efforts of art will, I apprehend, prove unavailing to arrest it, though as we are taught by Laennec, nature may sometimes interpose a shield to save the sufferer.

As a remedy in the local strumous affections, we are persuaded, there is none which deserves to rank with iodine. Its success in the treatment of white swellings is attested by a large number of cases, scattered through the periodical Journals of medicine within the last few years. Without entering into a detailed or elaborate statement of the experience of the profession generally, (for there is a striking coincidence in the results obtained by all experimentalists) we shall content ourselves with presenting that of Mons. Bayle, as indicating sufficiently the value of our medicine and the description of cases in which it promises benefit. In the Revue Medicale for March 1829, this gentleman reports twenty five cases of white tumours which were treated with iodine, some by himself, the rest by other physicians. The patients possessed lymphatic constitutions and had symptoms of scrophula. With the exception of one, who was in articulo mortis when the treatment commenced, they all improved remarkably in general health. Of the twenty-five, twenty-four were affected with tumours of the joints. In eight the tumour was situated in the knee, in six it occupied the foot, in three the hip, in two the elbow, and in the remaining five, the disease involved more than one articulation, and was accompanied with other symptoms of scrophula as ophthalmia, albugo, impetigenous ulcerations, &c. &c. They were all treated with iodine. To all, the tincture was administered in various doses, and in eight cases the ointment of iodine was used at the same time. Results.—Fifteen were cured, five were benefitted, four not improved. The cases cured, were those, in which the tumour was neither voluminous, nor open in many places, nor accompanied with atrophy of the limb, or general emaciation.

In behalf of our medicine in strumous ophthalmia, we might collect a very large amount of evidence, but to the readers of the Western Journal, we are persuaded, we can name no higher authority, than its distinguished senior editor whose ample experience, we are permitted to say, fully sustains the reports which have been made by others in favour of iodine in this disease. Our countryman, Dr. Cartwright of Natchez, has extended its employment to chronic ophthalmia accompanied with a turgid state of the vessels of the tunica conjunctiva lining the eye lids, to ophthalmia associated with what he calls the "*paludal diathesis*," (a term sufficiently indicative of the constitution he would designate) to iritis, and to other forms of ophthalmic disease. Whether in these it deserves any large share of consideration, remains to be ascertained by the observations and research of future enquirers. At present its value in these cases, rests almost exclusively upon his solitary experience.

As an emmenagogue, iodine has been used with a success which entitles it to the character of a valuable auxiliary to our resources in the treatment of amenorrhœa. The cases to which it has appeared to be best adapted, are such as are usually accompanied with vicarious hœmorrhage, such as are associated with enlargements of the spleen or liver, and are engrafted upon the paludal or strumous diathesis.

Leucorrhœa is another female complaint which has been made to succumb to the curative powers of our medicine. The experiments of Gimilbe and of Gœden in Europe would teach us that it is more especially suited to the "Leucorrhœa of habit" and to such cases as are dependent upon chronic irritation, but the trials of American physicians demonstrate that it is equally suited to all cases occurring in persons of a leucophlegmatic habit.

In chorea and paralysis iodine is highly commended by Manson, but his testimony is in some degree countervailed by that of Bardsley, who declares that he has never seen any benefit result from it in the latter disease, nor in the former, except in two cases. In the course of the experiments of the first physician, it occurred to him, to see fistula lachrymalis, combined with paralysis, removed by our medicine. A similar combination of disease presented him an opportunity of witnessing the cure of deafness by the same agent, and his further trials with it, have furnished eight other cases, as monuments of its utility in this disorder. It is probable that the disease in all these instances was occasioned by tumours pressing upon the membranous portion of the eustachian tube.

Enlargements of the liver and of the spleen have yielded to iodine, and upon the authority of Dr. Milligan of the Royal Universal Infirmary for children in London, it is stated that in one case of enlarged spleen it proved successful, after all the ordinary preparations of mercury had been unavailingly tried. Associated with, or consequential to this condition of the liver, and of the spleen too, it is not unusual to meet with cases of ascites. In this form of dropsy, thus complicated, our medicine has proved powerfully diuretic and efficacious in the hands of Bardsley and of Baron. In all the instances which have been detailed by the former physician, the patient laboured under great debility and a feeble pulse. In the reduction of indurated and enlarged testicle, iodine has been successfully employed by Dr. Deselleic of Paris, and by Drs. Cartwright, Thompson and M'Pheters of this country. It is our duty, however, to state that the same fortunate results have not been obtained by others. Our friend, Dr. Drake, informs us that he employed it without any benefit whatever, in a case of enlarged and irritable epidydymis, associated with dyspeptic symptoms. We would also add, that he has not, in the course of very extensive experiments with the article, observed it to diminish the size of the healthy testicle, as it has been reported to do.

While upon this branch of our subject (viz. glandular and visceral enlargements) we cannot refrain from calling the attention of the reader to a most interesting, though solitary case of enlarged uterus, detailed by Dr. Thitford of Ireland, in the transactions of the King and Queen's college of physicians. The patient had suffered very much in her general health for years. The whole substance of the uterus was discovered to be enormously distended and indurated, possessing the hardness of bone. She was subjected to treatment by mercury, conium and hyosciamus for 36 days, with some little improvement in her general symptoms, but without any effect whatever, upon the tumour. At the end of this time the tincture of iodine was substituted, and the tumour was quickly absorbed. Inflammation of this viscus, succeeding parturition, has been also cured in two instances, by Dr. Guerard, with iodine.

Acting upon the supposition, that every morbid growth of the body, wherever situated, presented a fair case for the trial of iodine, Dr. Gendrin has employed the ointment of the hydriodate of potash *successfully*, to promote the absorption of arthritic concretions ; and by a legitimate process of reasoning, which presumed that whatever acted favourably upon these would likewise benefit the disease itself, he has introduced it into the treatment of the paroxysms of Gout. His practice has already furnished numerous cases of its success ; (26 we have seen recited) but as far as we are informed, he has not been followed by any other experimentalist in this disease. Lately, Mr. Maury, of the hospital at St. Louis, in Paris, has succeeded with our medicine in curing two cases of rheumatism which had resisted all the usual remedies. It promptly dispersed the tumefaction of the joints. It has been used also in chronic ulcerations, in diseased heart, in cephalalgia, in obesity and in fungus hæmatodes ; but its value in all of these diseases is problematical, and with the exception of the first, rests upon a single case of success, which is, moreover, in the last countervailed by an instance of failure.

We have thus enumerated succinctly, and without regard to nosological arrangement, the principal diseases in which iodine has been used. Could we have designated also the circumstances of the patient, and the symptoms of each case, in which it has been found useful, we should have supplied a most important desideratum in regard to it, and have given additional interest and value to our summary. But neither our own experience [illegible]r the meager and scanty reports to which we have had access enable us to engage in the attempt, with any prospect of advantage. For the present we must content ourselves with general facts, leaving it to the tact and sagacity of each individual practitioner to make the application for himself. More extensive experiments and more careful observation, are yet necessary to establish the sensible effects of our medicine upon the system and to connect them with its therapeutic agency. Within the range of its salutary action iodine has not been observed to produce any physiological changes in the system commensurate with its great and multifarious remedial powers. An increase of appetite and of the pulse, an unusual giddiness and a peculiar itching, are the most common effects of the medicine, but we have superadded to these by some foreign physicians, a direful catalogue of appalling symptoms, such as great acceleration of the pulse, heat and irritation of the fauces, pain in the orbits of the eyes with derangement of vision, pain of the internal ear and soreness of the gums with occasional salivation, head-ache, restlessness, watchfulness, atrophy of the mammæ, and of the testicle, voracious appetite, emaciation, pains in the chest, stomach and bowels, with vomiting and diarrhœa, tremors, palpitations of the heart, convulsions, cramps or palsy of the lower extremities and finally anorexia and death. This pompous array of the formidable effects of iodine produced an interdiction of its use by the governments of some of the Swiss Cantons, and excited a professional dread of it, which is scarcely yet removed by the multiplied proofs of its safety. It has hence been forced to make its way through much opposition and indiscriminate abuse. The experience of Coindet, DeCarro, Breschet, Formey, Coster, Kolley, Erlinger and others on the continent of Europe, who have employed it extensively without any bad effect whatever, sustained as it is by the universal experience of British and American practitioners, should be considered as decisive of the question of its safety. Like mercury and arsenic, we believe, it is capable of exerting a poisonous influence upon the system, but it can be no more necessary to induce this influence, in order to attain its salutary action, than it would be to produce an exfoliation and destruction of the bones or a mercurial eczema in order to obtain the good effects of mercury in any of the diseases in which we habitually employ it. A prudent and skilful physician will always watch the effects of all potent medicines and continue, suspend or renew their use accordingly as the system may be impressed by them, and he will be thus often rewarded with a success which the timid, routine practitioner may in vain aspire to.

The contra-indications of our medicine, like its deleterious properties have doubtless been exaggerated, and unnecessarily multiplied. The mischievous effects which have been said to result from its employment, have inspired a professional timidity which is unfavourable to correct observations ; and many of the restrictions which have been imposed upon its use, we believe, are founded upon hypothetical grounds, rather than upon experience. It is said to produce unpleasant effects upon nervous and irritable habits and hence should be used with great caution in persons distinguished by this temperament. It nevertheless not unfrequently displays its *salutary action more readily in the old and infirm.* An unhealthy state of the digestive organs has been also supposed to furnish a contra-indication to its use, so have a disposition to phthisis pulmonalis, diarrhœa, local affections of the stomach and liver ; but we have seen that *many of these diseases* have been occasionally successfully treated by it, and *of course* they can now no longer be regarded as pathological conditions forbidding its employment. Its interdiction, in phthisical patients, is based most probably upon the supposition, long entertained, of its identity with the active principle of burnt sponge, which we are told by Hufeland is mischievous in such subjects. The chemical investigations of Heusmans of France, have demonstrated however, that it is not contained in burnt sponge. But if we wanted further evidence of the partial observation in which this idea originated, we need only refer to the extensive clinical experience of Lugol, of Berton and of Baron. The existence of inflammatory or congestive disorders, a full, plethoric habit and an accumulation of sordes in the alimentary canal are the only conditions of the system which, *in our opinion*, indisputably contra-indicate iodine, and they should always be removed by their appropriate remedies previously to its use.

